# Genome-wide identification and expression analysis of *auxin response factor* gene family in *Medicago truncatula*

**DOI:** 10.3389/fpls.2015.00073

**Published:** 2015-02-24

**Authors:** Chenjia Shen, Runqing Yue, Tao Sun, Lei Zhang, Luqin Xu, Shuanggui Tie, Huizhong Wang, Yanjun Yang

**Affiliations:** ^1^College of Life and Environmental Sciences, Hangzhou Normal UniversityHangzhou, China; ^2^Henan Academy of Agricultural SciencesZhengzhou, China; ^3^Department of Plant Pathology, Washington State UniversityPullman, WA, USA

**Keywords:** auxin response factor (ARF), auxin, auxin signaling, nodule formation, *Sinorhizobium meliloti* infection

## Abstract

Auxin response factors (ARFs) bind specifically to auxin response elements (AuxREs) in the promoters of down-stream target genes and play roles in plant responses to diverse environmental factors. Using the latest updated *Medicago truncatula* reference genome sequence, a comprehensive characterization and analysis of 24 *MtARF* (*M. truncatula ARF*) genes were performed. To uncover the basic information and functions of *MtARF* genes during symbiosis, we analyzed the expression patterns of *MtARF* genes during the early phase of *Sinorhizobium meliloti* infection. The systematic analysis indicated that changes in *MtARF* gene expression occur during these early stages of infection, suggesting a functional role in symbiosis. Furthermore, the roles of MtARF-mediated auxin signaling in symbiosis were tested in the infection resistant mutant (*dmi3*). The expression responses of *MtARFs* to *S. meliloti* infection were attenuated in the mutant compared to wild-type A17. In summary, our results show that changes in *MtARF* gene expression occur during the response to *S. meliloti* infection, suggesting that members of this family may have important roles in the symbiotic interaction.

## Introduction

The phytohormone auxin is involved in regulating many aspects of plant growth and development, including root system architecture re-establishment under nutrient stress and responses to environmental stimuli. The transcription of numerous genes are altered rapidly and specifically by auxin treatment (Chung et al., 2011; Molesini et al., 2014) and the *Auxin Response Factor* (*ARF*) gene family plays a vital role in response to indole-3-acetic acid (IAA) by regulating expression of down-stream target genes. Many early auxin-responsive gene promoters contain one or several copies of a conserved motif (TGTCTC) or its variants, which is called the auxin-responsive element (AuxRE) (Guilfoyle and Hagen, 2007). Components of the auxin signaling pathway have already been revealed in recent years (Hayashi, 2012). Recognition of the auxin/indole-3-acetic acid (Aux/IAA) proteins by the auxin transport inhibitor response 1 (TIR1), which is located at the top of SCF^TIR1/AFB^ ubiquitin ligase, accelerates Aux/IAA protein degradation by the 26S proteasome. Therefore, ARFs, which are inhibited by Aux/IAA, are released and began to modulate the expression of their target genes (Tan et al., 2007).

Classical genetic approaches have been used to functionally characterize the *ARF* genes during plant growth, development, and responses to environmental stimuli. In *Arabidopsis*, the *ARF3* loss-of-function mutation caused a defect in gynoecium patterning (Nishimura et al., 2005); the *arf5* mutant showed abnormal formation of vascular strands and the embryo axis (Hardtke and Berleth, 1998); the *ARF7* loss-of-function mutation impaired hypocotyl response to blue light and auxin stimuli (Harper et al., 2000); the *ARF8* loss-of-function mutation affected hypocotyl elongation and auxin homeostasis (Goetz et al., 2006) and a double mutant, *arf7/arf19*, affected auxin mediated lateral root development (Narise et al., 2010). Transgenic rice expressing an antisense of OsARF1 were stunted in growth, sterile, and had low vigor, curled leaves, as well as altered organ size, relative to wild-type plants (Aya et al., 2014). *OsARF12* has been shown to play a role in iron homeostasis (Qi et al., 2012) and *OsARF16* facilitated the efficient utilization of phosphate in rice plant (Shen et al., 2013).

Many legume species interact with nitrogen-fixing bacteria (rhizobia) to form symbioses (van Noorden et al., 2007). Numerous genes are specifically involved in the formation of nodules, which are the symbiotic legume organs that house nitrogen-fixing bacteria during host plant and symbiotic rhizobia interactions (Nallu et al., 2013). Previous reports indicated that there was a positive correlation between auxin signaling and nodule formation in *Medicago truncatula* (Roudier et al., 2003; Kondorosi et al., 2005). Auxin, which plays a crucial role in plant control of cell division and elongation, is likely to be an important nodulation regulator (Blilou et al., 2005). Auxin signaling is initiated through activation of transcriptional response mediated by the AUX/IAA and ARF families of transcriptional regulators (Chapman et al., 2012). In *Arabidopsis*, ARF2 was reported to be involved in the controlling of differential cell division and elongation, which was an important process of nodulation (Li et al., 2004). Furthermore, inhibition of auxin polar transport at the nodule site is a prerequisite for nodule initiation in indeterminate legumes (Wasson et al., 2006).

As a model indeterminate legume, *M. truncatula* has been used to reveal how auxin signaling participates in nodule initiation. A high concentration of auxin reduced the number of nodules in *M. truncatula* and a low concentration of auxin increased nodule numbers (van Noorden et al., 2006). Previous studies showed that in *M. truncatula* the expressions of the auxin responsive reporter genes were localized in the inner and outer cortical cells when they were dividing, which indicated that regulation of mitosis during nodulation was the main role of auxin (Kondorosi et al., 2005). The control of auto-regulation of nodulation (AON) depends on long-distance auxin transport from the shoots to roots (van Noorden et al., 2006). The application of auxin polar transport inhibitors, such as N-(1-naphthyl) phthalamic acid (NPA) and 2, 3, 5-triiodobenzoicacid (TIBA) induced the formation of pseudo-nodules in *M. truncatula* (Rightmyer and Long, 2011). A close relationship has been found between the root nodule formation and auxin transport inhibition (Wasson et al., 2006). However, the underlying mechanism linking auxin signaling and nodule formation during the early phase of *Sinorhizobium meliloti* infection of *M. truncatula* remains largely unknown.

As an important segment of the auxin signaling pathway, ARFs are encoded by a multigene family present in many different plant species. There are 23 members in *Arabidopsis*, 22 members in tomato (*Solanum lycopersicon*), 31 members in maize (*Zea mays* L.), 15 members in cucumber (*Cucumis sativus*), 39 members in poplar (*Populus trichocarpa*), 25 members in rice (*Oryza sativa* L.), and 51 members in soybean (*Glycine max* L.) (Guilfoyle and Hagen, 2007; Kalluri et al., 2007; Liu et al., 2007; Wang et al., 2007, 2012; Shen et al., 2013; Zouine et al., 2014). This study provides detailed information on the gene structures, chromosomal locations, sequence homologies, and expression patterns of 24 *ARF* genes in *M. truncatula*. We provide new information about the complexity of *M. truncatula ARF* expression control during the early phase of *S. meliloti* infection. The distinct spatio-temporal expression patterns for *M. truncatula ARF* genes and their differential responses to rhizobial symbiosis provide the foundation for a more comprehensive functional characterization of these transcriptional mediators and their involvement in the formation of root nodules.

## Materials and methods

### Plant material, growth conditions, and hormone treatment

*M. truncatula* cv Jemalong plants (wild-type A17) were scarified with sand paper, surface sterilized with 6.25% (v/v) hypochlorite for 8 min and washed five times with sterile water, and germinated on plates containing buffered nodulation medium BNM (Engstrom et al., 2002) with 0.8% agar and 1 μM imidazoline betaine (AIB) in the dark over night at 30°C. Seedlings were transferred to large Petri dishes containing a nitrogen-free BNM medium, five seedlings per plate. Plates were kept vertical and black paper was used to cover the sides of each plate, and an aluminum foil spacer was laid between the medium and Petri dish to help air exchange. Seedlings were incubated in a growth chamber at a constant 22°C during a 16 h day and 8 h night with a photon flux density of 100 μmolm^−2^s^−1^. For the infection treatment on *M. truncatula* seedlings, 14-day-old seedlings (10 seedlings) were soaked in liquid BNM medium with or without (mock treatment) 10 μM IAA, then the roots and shoots of *M. truncatula* seedlings were collected for RNA isolation. For infection experiment, the seedlings were soaked in liquid BNM medium under different treatments. These treatments were –Sin/–NOA, +Sin/–NOA, –Sin/+NOA, and +Sin/+NOA. The treatment –Sin/–NOA was used as mock treatment (Sin = *S. meliloti* infection; NOA = 10 μM 1-NOA treatment; –Sin/–NOA: mock treatment; –Sin/+NOA: NOA treatment positive control). The experiment was repeated for five times with similar results.

### Identification of ARF genes in *M. truncatula*

Comprehensive identification of *M. truncatula ARF* gene family members was achieved using *Arabidopsis* ARF proteins previously reported as phytozome BLAST queries of the *M. truncatula* genome database (http://www.phytozome.net/). The e-value acceptable in the Blast analysis for ARF member identification was “10^−3^”. The hidden Markov model (HMM) profiles of the *ARF* gene family [Pfam 02309: AUX/IAA family; Pfam 06507: ARF (AUX_RESP); Pfam 02362: B3 DNA binding domain (B3)] were employed to identify the *ARF* genes from the *M. truncatula* genome. These profiles were used to search the complete proteome of *M. truncatula* available in phytozome (http://www.phytozome.net/). All the obtained sequences were sorted as unique sequences for further studies. B3, AUX_RESP, and Aux/IAA domain were analyzed using InterProScan Sequence Search (http://www.ebi.ac.uk/Tools/pfa/iprscan/). The miRNA locations on the *MtARFs* were searched using PMRD data (http://bioinformatics.cau.edu.cn/PMRD/). Linear display of synteny blocks were retrieved from the SyMAP database (http://www.symapdb.org/projects/fabaceae/).

### Phylogenetic tree building and prediction of amino acid composition

Multiple sequence alignments were performed on the MtARF nucleotide sequences using ClustalW with the default parameters. The gap open penalty parameter is 15, gap extension penalty parameter is 6.66, and the weight matrix selected is “IUB” for DNA. The alignments were subsequently visualized using GeneDoc (http://www.nrbsc.org/gfx/genedoc/) as presented in Figure S1. Phylogenetic tree was constructed with aligned 24 *MtARF* nucleotide sequences, 23 *AtARF* nucleotide sequences, and 51 *GmARF* nucleotide sequences using MEGA5.1 (http://www.megasoftware.net/mega.php) employing the neighbor-joining (NJ) method. Bootstrap values were calculated from 1000 iterations. The constructed tree file was visualized by TreeView1.6 (http://taxonomy.zoology.gla.ac.uk/rod/treeview.html). The software MEGA 5.1 was used for calculation of amino acid composition of the MR domain in MtARFs.

### Intron/exon structure, genome distribution, and motif prediction

The DNA and cDNA sequences corresponding to each predicted gene from the *M. truncatula* genome and the information of *MtARFs* intron distribution pattern were obtained from the http://www.phytozome.net/cgi-bin/gbrowse/medicago. To obtain the gene location, we drew a map of the distribution of *MtARF* genes throughout the *M. truncatula* genome. Comparisons to the Pfam database were used to identify motifs present in the MtARF protein sequences.

### RNA isolation and qRT-PCR

Total RNA from the cotyledons, leaves, shoots, roots, and flowers was extracted using a Plant RNeasy Mini kit (Qiagen, Hilden, Germany) according to the manufacturer's instructions. Then DNase I treatment was used to remove genomic DNA contamination from total RNA. The cDNA was synthetized using ReverAid First Strand cDNA Synthesis Kit (Thermo Scientific #1622). The primer sequences are listed in Table S1. Briefly, 1 μl of a 1/10 dilution of cDNA in double distilled water was add to 5 μl of 2×UltraSYBR, 100 nM of each primer and water was then added to final volume 10 μl. The procedures for PCR were as follows: 95°C for 10 min; 40 cycles of 95°C for 15 s, 60°C for 60 s. (All reactions were run in triplicate.) Relative fold differences were calculated based on the comparative *Ct* method using *Mt-Actin* (*MTR_2g008050*) as an internal standard. Heat map representation was performed using the normalized (2^−ΔΔCt^) value with ClustalW software and Treeview to visualize the data. The expression folds were used by Treeview to visualize as heat map. In our experiment, a specific fold change value (2×) in the expression levels is used to clarify the statistical analysis of significant differences among mock and the treatments. All the expression analysis was carried out for five biological repeats and the values shown in figures represented the average values of these five repeats. Three technical replicates were contained in each biological repeat.

### Bacterial strains and rhizobia infection

The rhizobia strain used for inoculating the roots of seedlings was *S. meliloti* strain 1021 (from ATCC data base, ATCC^®^ Number: 51124), a streptomycin-resistant derivative of wild-type. *S. meliloti* was grown overnight at 28°C in liquid LBMC medium (10 g/L tryptone, 5 g/L yeast extract, 10 g/L NaCl, 2.6 mM MgSO_4_, 2.6 mM CaCl_2_) supplemented with 200 μg/ml streptomycin, then collected by centrifugation, and suspended in 10 mM MgSO_4_. The bacterial suspension was diluted with liquid BNM medium to OD_600_ = 0.1. For plant inoculation, each seedling was placed in a single 50 ml tube containing the nitrogen-free nutrient solution plus perlite and treated with BNM medium diluted bacteria. For control (mock-inoculated), the seedlings were treated with sterilized BNM medium containing 10 mM MgSO_4_. Different sets of plants were grown and used for the various analyses.

## Results

### Genome-wide identification of ARF genes in *M. truncatula*

After gene structure and protein domain analysis, 24 *ARF* genes were identified in *M. truncatula*. These genes were named according to gene locations on the chromosomes (from Chr. 1 to Chr. 8). All the information on these 24 *MtARF* genes, such as gene names, locus IDs, ORF lengths, numbers of introns, locations on chromosomes, and basic information about deduced polypeptides are listed in Table S2. The sizes of the deduced MtARF proteins varied markedly from 323 amino acids (MtARF4) to 1252 amino acids (MtARF22). The corresponding molecular masses varied from 36.92 to 141.13 kDa and the predicted isoelectric points varied from 5.08 (MtARF16) to 8.48 (MtARF22).

### Chromosomal distribution and expansion analysis of *MtARF* genes

**Figure 1:**
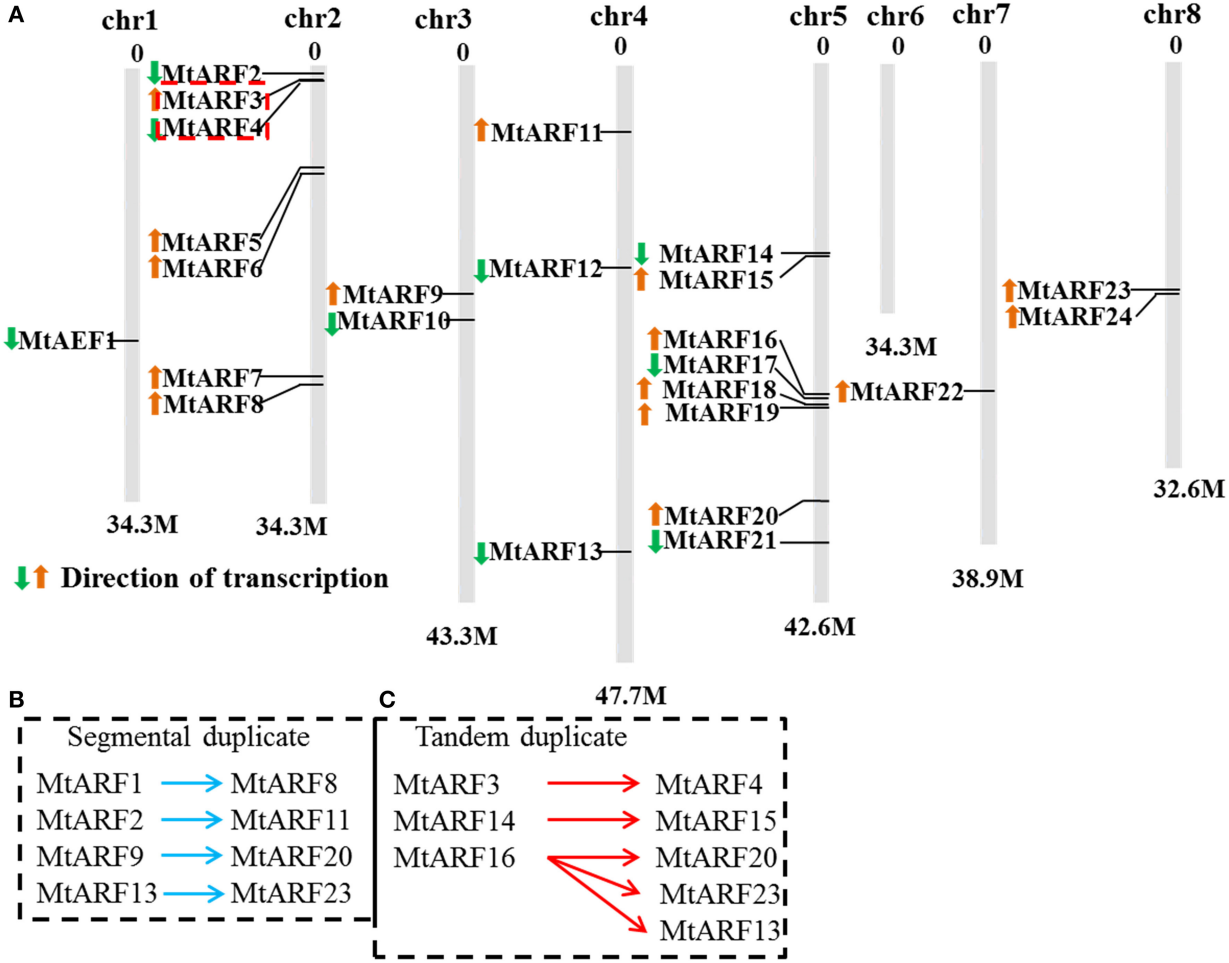
**Chromosomal distribution and expansion analysis of *MtARF* genes. (A)** Twenty-four *MtARF* genes were among eight chromosomes except chromosomes 6. Arrows in front of gene names indicated the direction of transcription. **(B)** Four pairs of segmental duplicates *MtARF1/MtARF8*, *MtARF2/MtARF11*, *MtARF9/MtARF20*, and *MtARF13/MtARF23* were linked by blue arrows. **(C)** Tandem duplicates *MtARF3/MtARF4*, *MtARF14/MtARF15*, and *MtARF16-19* were limited by red arrows.

*MtARF* gene locations were mapped on chromosome sequences in order to gain an insight into the organization of *MtARF* genes, which may be involved in auxin signaling. Based on the available *M. truncatula* genome assembly sequence, the *M. truncatula ARF* gene mapping revealed that 24 *MtARF* genes were distributed among seven chromosomes (out of a total of eight chromosomes in the *M. truncatula* genome). A total of 24 *MtARF* genes were present on all chromosomes, except chromosome 6. Among these 24 *MtARF* genes, seven (29.2%) genes were located on chromosome 2 and eight (33.3%) genes were located on chromosome 5. Chromosomes 1 and 7 contained only one *MtARF* gene each; chromosomes 3 and 8 each contained two *MtARF* genes and chromosome 4 had three *MtARF* genes (Figure 1A).

Many studies have found that gene divergence and duplication events are the primary contributors to evolutionary momentum (Bowers et al., 2003). Gene duplication events, which consist of tandem and segmental duplications, were identified and plotted on to chromosomes in an attempt to explain the expansion of *M. truncatula ARF* family genes during the evolutionary process. All gene sister pairs that were classified into tandem duplication or segmental duplication groups were shown in Figures 1B,C. Seven gene sister pairs were identified. These were four pairs of segmental duplicates: *MtARF1/MtARF8*, *MtARF2/MtARF11*, *MtARF9/MtARF20*, and *MtARF13/MtARF23*, which are linked by the blue arrows, and three tandem duplicates: *MtARF3/MtARF4*, *MtARF14/MtARF15*, and *MtARF16-19*, which are delimited by the red arrows.

### Analysis of amino acid composition and classification of MtARFs

The domain positions in the 24 MtARF proteins are shown in Table S3 and the amino acid composition of the Middle Regions (MRs) is shown in Figure S2 (the data was listed in Table S4). The 24 MtARFs were categorized into three subgroups, based on their MR amino acid composition and the presence or absence of Carboxy-Terminal Domains (CTDs). These are: (1) ARF proteins with a DNA Binding Domain (DBD), activator MR and a CTD; (2) ARF protein with a DBD, repressor MR and a CTD; and (3) ARF proteins with a DBD, repressor MR, but no CTD (Figure S2B). The *M. truncatula* ARF family encodes five putative transcriptional activators: MtARF6, 9, 13, 20, and 24 [MR enriched with glutamine (Q), serine (S), and leucine (L)] and five putative transcriptional repressors: MtARF2, 7, 8, 22, and 23 [MR enriched with S, L, proline (P) and glycine (G)]. Fourteen MtARF proteins are putative transcriptional repressors that do not contain a CTD: MtARF1, 3, 4, 5, 10, 12, 14, 15, 16, 17, 18, 19, and 21.

### Analysis of phylogenetic relationships among ARF proteins

**Figure 2:**
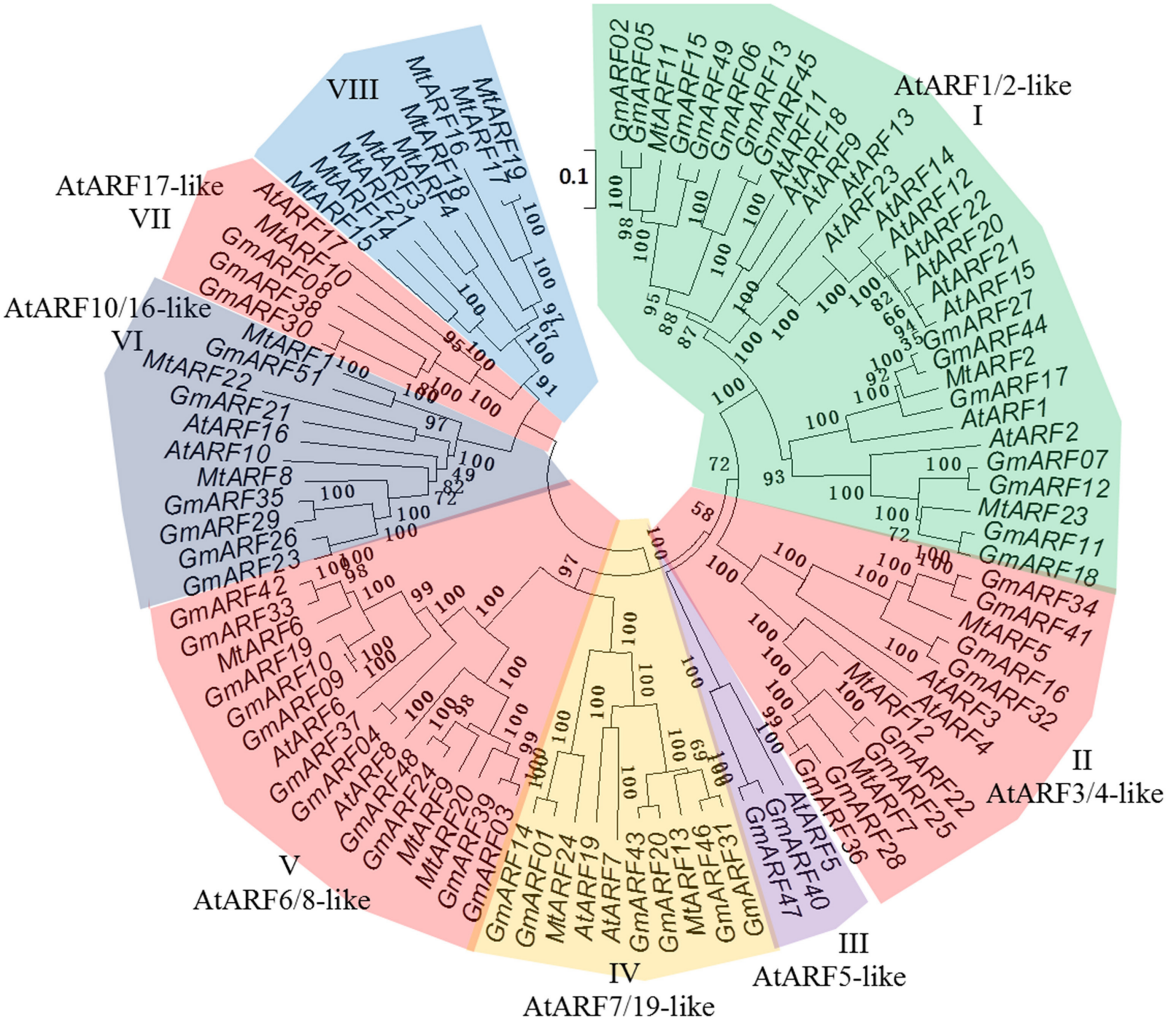
**Analysis of phylogenetic relationship of *ARF* genes in *M. truncatula*, model plant *Arabidopsis* and leguminous plant soybean.** Bootstrap values are presented for all branches. Twenty-four *MtARF* genes, 23 *AtARF* genes, and 51 *GmARF* genes were classified into eight subclasses (from I to VIII). Scale bar 0.1 denoted 0.1 nucleotide substitution per site. The *ARF* genes were color coded according to their classes.

A phylogenetic tree was constructed to explore the phylogenetic relationships of *ARF* genes among different species. The ARF genes in the model plant *Arabidopsis* and leguminous plant soybean were used for phylogenetic tree construction (Figure 2). The phylogenetic distribution revealed that *ARF* genes can be grouped into eight major subclasses (from I to VIII). All the MtARFs that were predicted to function as transcriptional activators and contained a Q-rich MR domain belonged to subclasses IV and V (MtARF6, 9, 13, 20, and 24) and MtARFs that were predicted to function as transcriptional repressors belonged to subclass I (MtARF2, 7, 8, 22, and 23). Most of the MtARF proteins containing the B3 DNA binding domain (DBD) and the AUX/IAA family domain (CTD) were found in subclass I, II, IV, and V (MtARF1, 5, and 12 were belonged into subclass II. All the MtARFs in subclass VI, VII, and VIII did not contain an AUX/IAA family domain (CTD). The *MtARF* genes from subclass I, II and IV–VII were very closely related to the *ARF* genes from soybean and *Arabidopsis*. However, the *MtARF* genes belonged to subclass VIII, were not closely related to any soybean or *Arabidopsis ARF* genes indicating a diverging trend in the evolution of ARF family members across different plant species.

### Expression patterns for *MtARF* genes in different *M. truncatula* tissues

**Figure 3:**
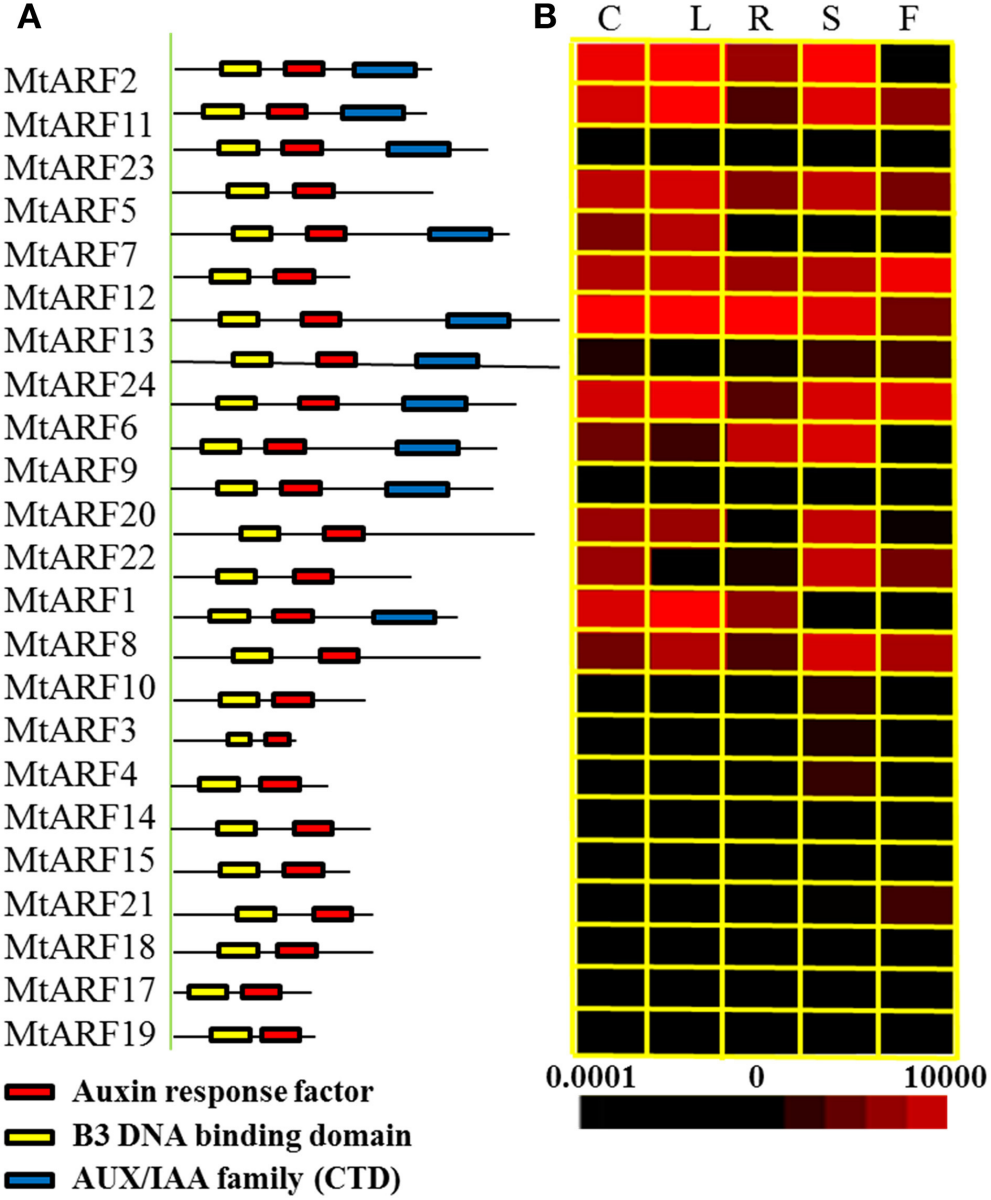
**Analysis of protein domains and tissue-specific expression patterns. (A)** Depiction of the domain structure of the ARF protein sequences. The auxin response factor domain, B3 DNA binding domain and AUX/IAA family domain are colored in red, yellow, and blue, respectively. **(B)** Heat map representation for tissue-specific expression patterns of 24*MtARF* genes. C, cotyledon; L, leaf; R, root; S, shoot; F, flower. Expression patterns of the *MtARF* genes in five indicated tissues were determined using qRT-PCR. The different colors correspond to the values of the gene expression compare to *MtACTIN* shown in the bar at the bottom of figure. The color scale of each dendrogram represents expression values; black indicates a low level and red represents a high level of transcript abundance. The expression level of *MtACTIN* was defined as 1 × 10^4^.

The spatial specificity expression pattern of individual members of the *MtARF* family was examined in the different *M. truncatula* tissues using qRT-PCR. These were the roots, stems, cotyledons and leaves of 2-week old seedlings and the flowers from 2-month old plants. Transcripts of the 24 *MtARF* genes could be detected in the different tissues (Figure 3). Most *MtARF* gene expressions were ubiquitously in all tissues, which suggested they have a putative function in many aspects of plant growth and development, including root system regulation, shoot initiation and growth, apical dominance, and embryonic development.

### Prediction of the microRNA putative target sites of *MtARF* genes

In *Arabidopsis*, the expression levels of *ARF6* and *ARF8* are regulated by *microRNA167* (Yang et al., 2006) and *ARF10*, *16*, and *17* were targeted by *microRNA160* (Liu et al., 2007). To explore whether there was potential regulation of *MtARF* genes by microRNA, we studied the ARF protein phylogenetic relationships between *Arabidopsis* and *M. truncatula*. A phylogenetic tree was constructed using ARF family members from *Arabidopsis* and *M. truncatula*. The results showed that *MtARF1* and *MtARF10* were highly homologous with *AtARF16* and *AtARF17*, and *MtARF6* and *MtARF9* were highly homologous with *AtARF6* and *AtARF8*, respectively.

**Figure 4:**
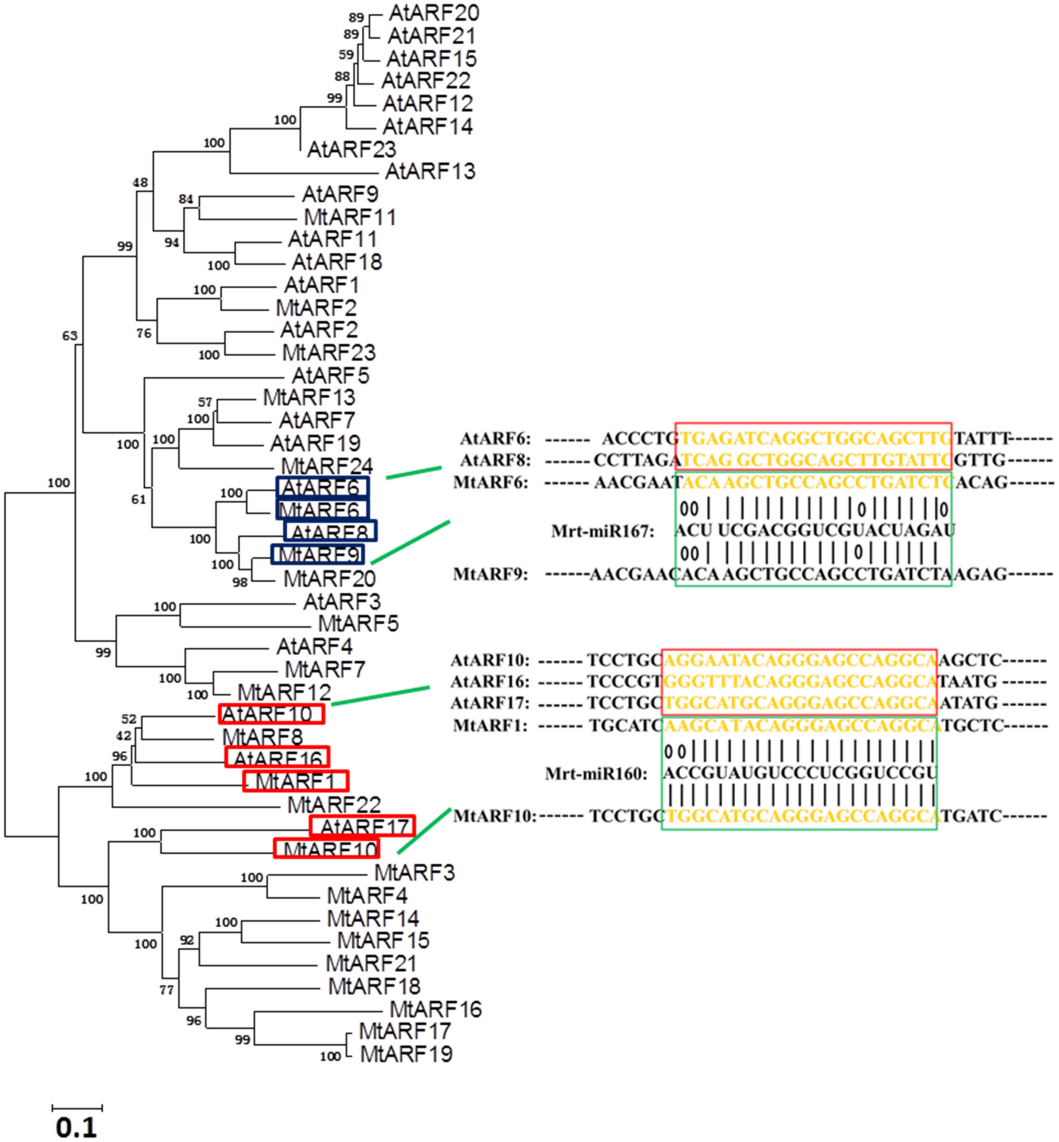
**Predictions of target sites of *Mrt-miR167* and *Mrt-miR160* in *MtARF* genes.** A phylogenetic tree was constructed that included ARF family members from Arabidopsis and *Medicago truncatula*. Database (PMRD) (http://bioinformatics.cau.edu.cn/PMRD/) was used to search the sequences of *Mrt-miR160* and *Mrt-miR167*.

Our results suggest that five precursors (*mtr-miR160, 160b, 160c, 160d, and 160e*) in the *M. truncatula* genome were needed to produce the mature *mtr-miRNA160* sequence (UGCCUGGCUCCCUGUAUGCCA), but only one precursor (*mtr-miRNA167*) was needed to produce the mature *mtr-miRNA167* sequence (UGAAGCUGCCAGCAUGAUCUA). Sequence analysis of *ARFs* in *M. truncatul*a implied that the 1344–1364 bp region on *MtARF1* and the 1314–1330 bp region on *MtARF10* may be the target sites of *miRNA160* and the 2457–2477 bp region on *MtARF6* and the 2301–2321 bp region on *MtARF9* may be the target sites of *miRNA167* (Figure 4).

### Expression responses of *MtARF* genes to auxin treatment in different tissues

As a key component of the auxin signaling pathway, responsiveness to auxin is one of the important features of *ARF* genes. In our study, the transcript levels of 24 *MtARF* genes were assessed by qRT-PCR in the shoots and roots of 2-week old seedlings after treatment with 10 μM IAA for 1 h. Most of *MtARF* genes were found to be auxin responsive after IAA treatment, with 14 *MtARF* genes showing up-regulation, whereas only *MtARF4, MtARF15, MtARF18*, and *MtARF21* displayed down-regulation in the shoots. *MtARF7*, *9*, *13*, *17*, and *24* showed almost no response to IAA treatment in the shoots. In the roots, the expression levels of 10 *MtARF* genes were up-regulated and 14 *MtARF* genes were down-regulated by IAA treatment. In the roots, *MtARF8* and *MtARF18* were strongly up-regulated (more than 10-fold increases) and *MtARF1*, *MtARF9*, *MtARF10* and *MtARF16* were sharply down-regulated after IAA treatment.

**Figure 5:**
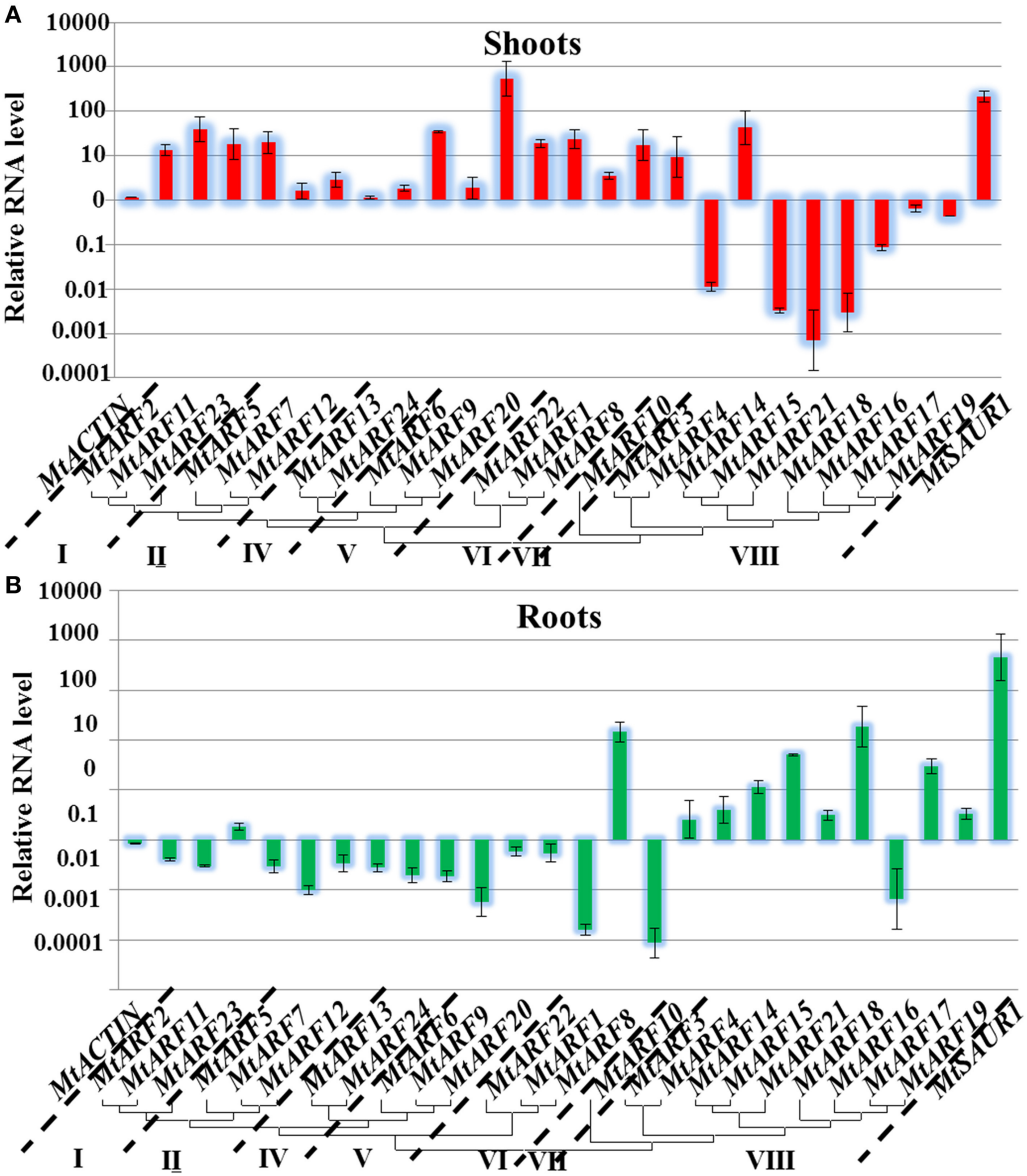
**Analysis of *MtARF* genes expression in response to auxin treatment.** Total RNA was extracted from the seedlings shoots **(A)** and roots **(B)** tissues of *M. truncatula* for basal expression. The histogram shows the relative expression level of *MtARF* genes under IAA treatment compared to the mock expression level. The relative mRNA level of individual genes was normalized with respect to the *MtACTIN* gene. Data from five independent replications were used in the analysis, and standard deviations were shown with error bars.

The expression for many of the *M. truncatula* ARF genes exhibited opposite patterns in shoots versus roots. The expression levels of 10 *MtARF* genes that were induced by IAA treatment in the shoots were actually down-regulated in the roots and the expression levels of five *MtARF* genes were down-regulated in the shoots after IAA treatment, but induced in the roots. The most down-regulated *MtARF* genes in the roots, *MtARF1* and *MtARF10*, were all strongly induced in the shoots after IAA treatment [over 10-fold increase, log (expression levels) > 1] (Figure 5).

### Expression analysis of *MtARF* genes during the early phase of *S. meliloti* infection

**Figure 6:**
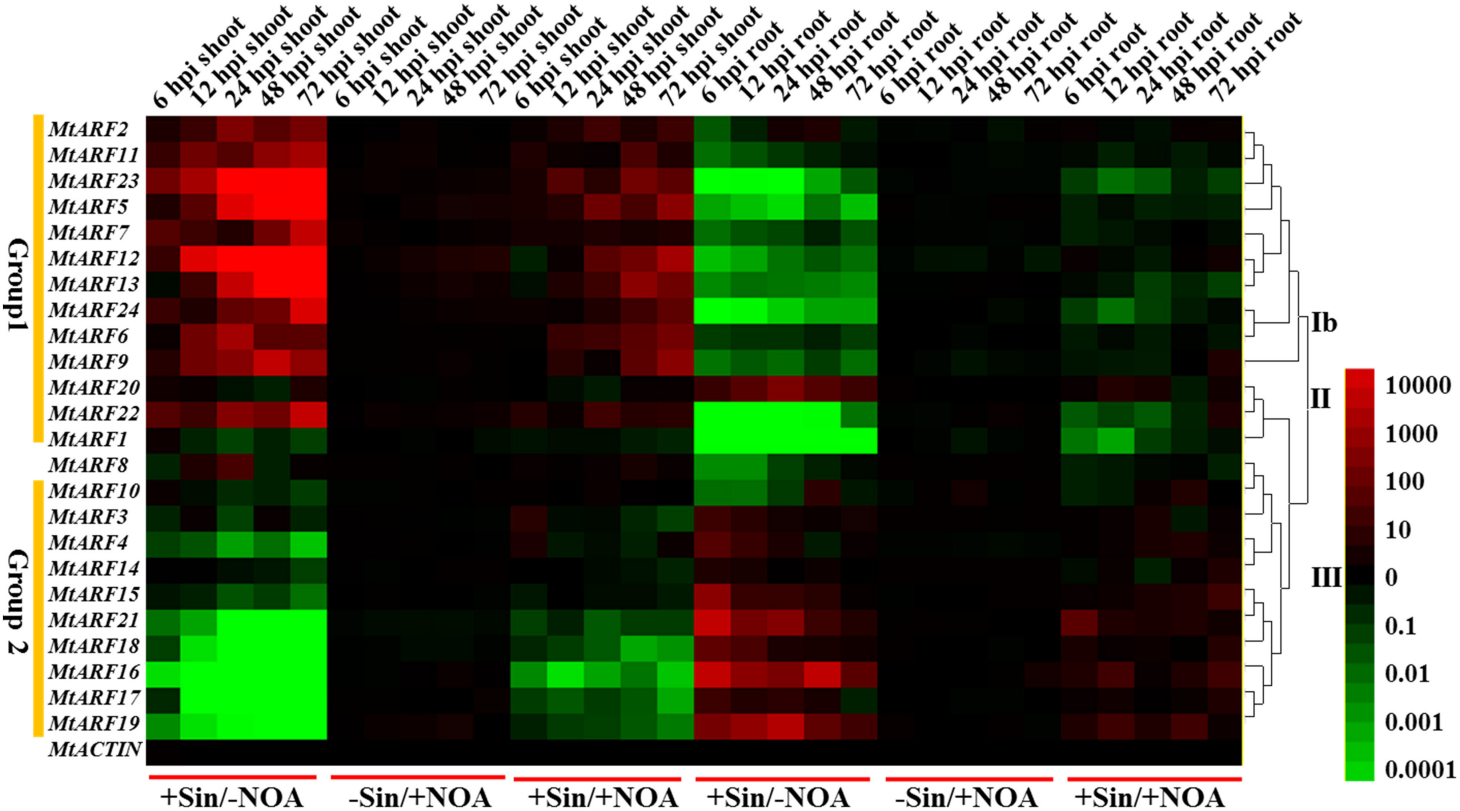
**Heat map showing *MtARF* gene expression patterns at the early phase of *S. meliloti* infection under different conditions.** Samples of two different tissues (shoots and roots) were used to test the changes of *MtARF* gene expression levels at different time points (6, 12, 24, 48, 72 hpi) and conditions (–Sin/–NOA, +Sin/–NOA, –Sin/+NOA and +Sin/+NOA). The data of –Sin/–NOA was used as control. The different colors correspond to the values of the gene expression changes shown in the bar at the right of figure. The red color represents up-regulation expression, black represents unchanged expression level and green color represents down-regulation expression.

To examine change in gene expression of the *MtARFs* genes during the early stage of rhizobial infection, we compared the *MtARF* gene expression profiling in the roots and shoots of *S. meliloti*-inoculated *M. truncatula* plants with those of mock-inoculated ones over a 72 h post-inoculation (hpi) period. Many gene expression changes related to the host responses occurred during the pre-infection or initial infection periods (Molesini et al., 2014), so different sampling time points (6, 12, 24, 48, and 72 hpi) were chosen for gene expression level analysis. 1-NOA that binds to auxin-influx transporters to block auxin polar transport was used to suppress auxin signaling. Mock-inoculated and bacteria-inoculated samples were separated into two different groups for both the root and shoot relative RNA level data sets (Figure 6).

Two main patterns for *MtARF* gene expressions were observed during the early phase of *S. meliloti* infection. Expression levels of *MtARF2*, *5*, *6*, *7*, *9*, *11*, *12*, *13*, *23*, and *24* (Group 1) were similar and were induced during the early phase of *S. meliloti* infection in the shoots and reduced in the roots. The expression levels of *MtARF3*, *4*, *10*, *14*, *15*, *16*, *17*, *18*, *19*, and *21* (Group 2) were opposite to genes in Group 1 in that transcript levels of all these genes showed a drastic decline during the early phase of *S. meliloti* infection in the shoots. The duration of the post-infection period after bacteria-inoculation had a particularly significant effect on the expression regulation of *MtARF* genes in the shoots. From 6 to 72 hpi, the changes in gene expressions increased notably in the shoots as the time after inoculation increased. In contrast, the opposite occurred in the roots.

In order to study the effect of *S. meliloti* inoculation on the expressions of MtARF genes, the expressions of *MtARF* genes were determined with or without 10 μM 1-NOA treatment after *S. meliloti* infection. Our data showed that the changes in *MtARF* gene expression levels were alleviated by 10 μM 1-NOA application. With 1-NOA treatment, the expression levels of Group 1 genes were down-regulated and expression levels of Group 2 genes were up-regulated in the shoots compared to the 1-NOA free treatment. In the roots, the 1-NOA treatment induced the expressions of Group 1 genes, but down-regulated the expressions of Group 2 genes compared to the 1-NOA free treatment.

**Figure 7:**
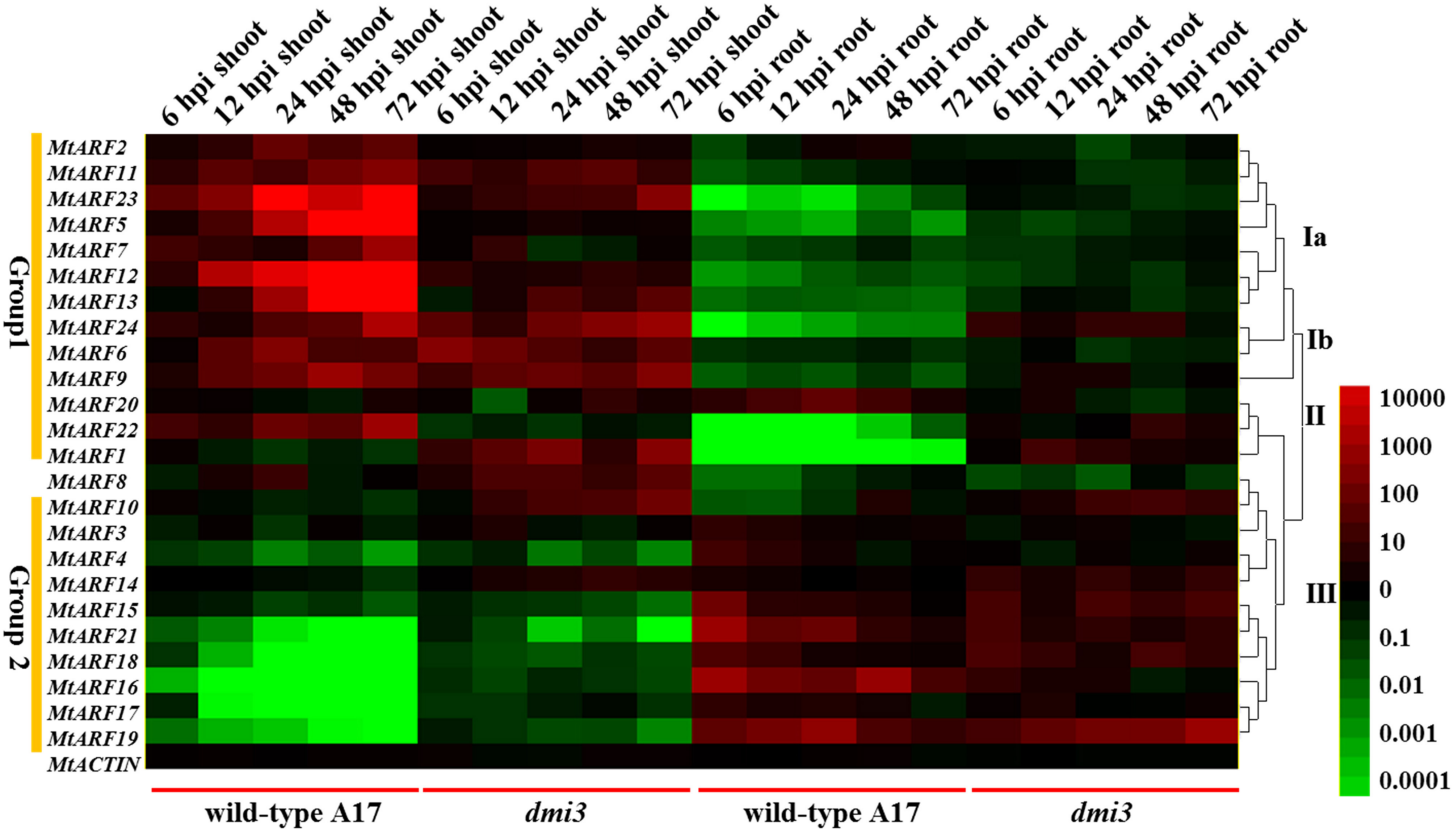
**Heat map showing *MtARF* genes expression pattern at the early phase of *S. meliloti* infection in wild-type A17 and mutant *dmi3*.** Samples of two different tissues (shoots and roots) were used to test the changes of *MtARF* genes expression level at different time points (6, 12, 24, 48, 72 hpi). The different colors correspond to the values of the gene expression changes shown in the bar at the right of figure. The red color represents up-regulation expression, black represents unchanged expression level and green color represents down-regulation expression.

Furthermore, we tested the possible involvement of MtDMI3 (does not cause infections, a *M. truncatula* gene that is necessary for infection by *S. meliloti*), a Ca (2+)/calmodulin-dependent protein kinase (CCaMK) that is essential for both arbuscular mycorrhizal (AM) and rhizobial symbioses (Messinese et al., 2007; Chen et al., 2008). In our study, the *dmi3* mutant was used to test the expression pattern changes in this infection resistant mutant during the early phase of *S. meliloti* infection. Our data showed that the expression levels of the *MtARF* genes during *S. meliloti* infection in the *dmi3* mutant were different to the wild-type A17 (Figure 7). The *DMI3* mutation reduced the differences in expression responses to *S. meliloti* infection. In contrast to the wild-type A17, it is implied that the *dmi3* mutant attenuated auxin signaling and reduced the differences in *S. meliloti* infection responses between the Group 1 genes and the Group 2 genes. The expression levels of Group 1 genes in the *dmi3* mutant were down-regulated and the expression levels of Group 2 genes in the *dmi3* mutant were increased in the shoots compared to the wild-type A17. In the roots, the *DMI3* mutation led to the increases of Group 1 genes and reductions in the expression of Group 2 genes compared to the wild-type A17.

### Analysis of the auxin-response *cis*-element in the rhizobial infection response gene promoters

Most auxin response gene promoters contain successive auxin-response elements (AuxRE), which are targeted by auxin-response factor (ARF) transcription factors at the transcription level (Sakamoto et al., 2013). The AuxRE, some bZIP and MYB transcription factor families are also involved in auxin transcriptional regulation *via* bZIP Response Elements (ZREs) and Myb Response Elements (MREs) (Berendzen et al., 2012). We examined the promoters of 48 rhizobial-responsive genes for DNA motifs that correlate with auxin regulation (Ortu et al., 2012). Using Ortu's microarray data (2012), 48 rhizobial infection response genes were chosen for promoter auxin-response *cis*-element analysis.

**Figure 8:**
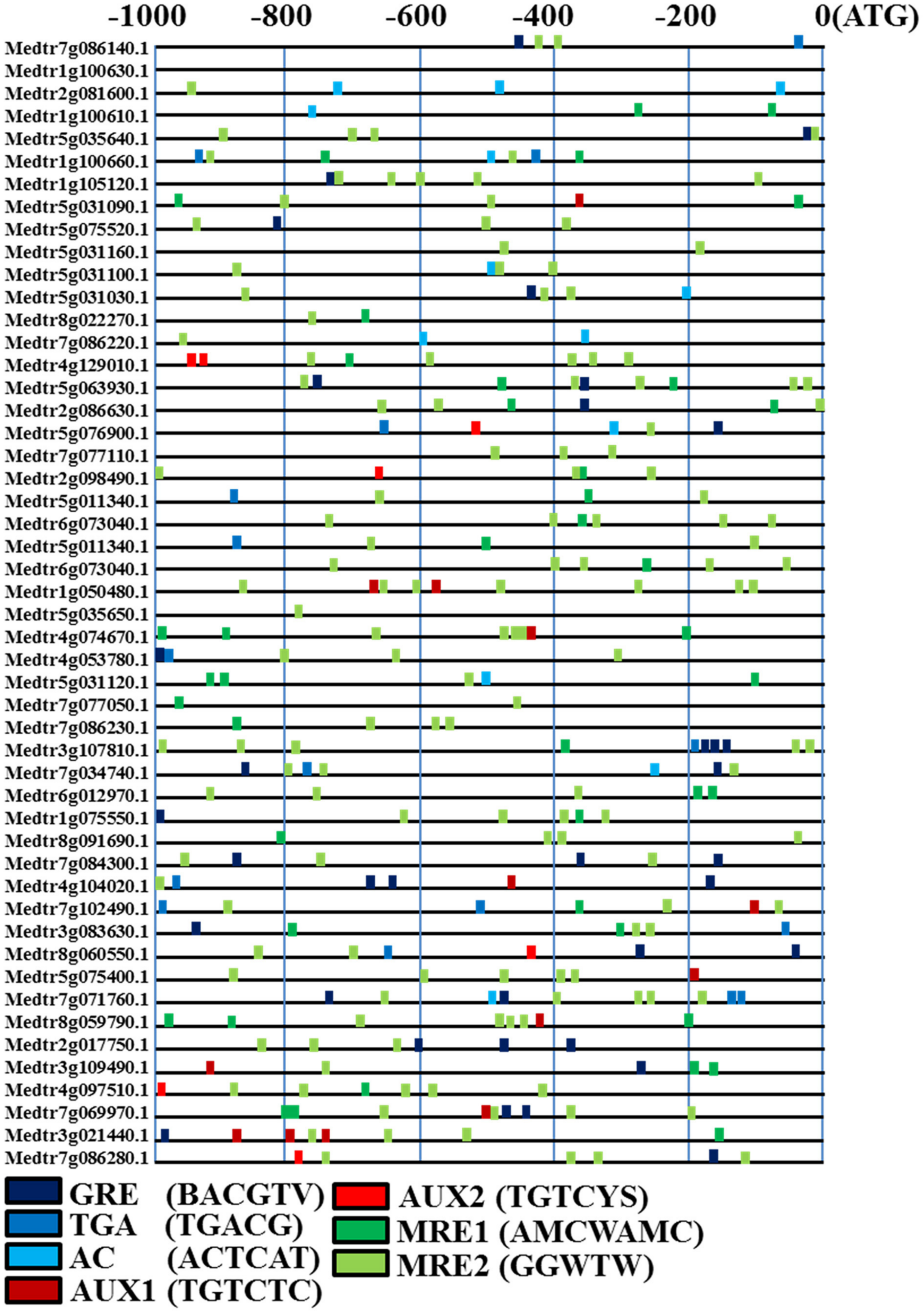
**Motif analyses of specific *cis*-elements in rhizobial infection response gene promoters from *M. truncatula*.** The 1000 nucleotides upstream from annotated start codons of differentially expressed were analyzed for the presence of sequences similar to ZRE, AuxRE, and MRE *cis*-elements, which are given using the presented color code. AUX1 is TGTCTC; AUX2 is TGTVYS, three different ZREs (GRE: BACGTV; TGA: TGACG; AC-motif: ACTCAT) and two MREs (MRE1: AMCWAMC; MRE2: GGWTW).

The promoters (−1000 to −1 bp) of rhizobial infection response genes were scanned for AUX1 (TGTCTC core sequence), a less stringent variant called AUX2 (TGTVYS), three different ZREs (GRE, TGA, and AC-motif) and two MREs (MRE1: AMCWAMC and MRE2: GGWTW). The motifs for AuxREs, ZREs, MREs, and MYC2 related elements were more abundant in the promoters of the rhizobial infection response genes (Figure 8). The MRE2 motif was highly frequent at more than twice the levels per 1000 bp promoter region (MRE2: 2.85/1000 bp) as the other genes. GRE, TGA, AC, AUX1, AUX2, and MRE1 were also present at relatively high levels. These data indicated that many rhizobial infection response genes could be regulated by ARF-mediated auxin signaling.

## Discussion

As an important component of the auxin signaling pathway, ARFs directly bind to and regulate the specific expressions of down-stream target genes during the auxin response process (Cho et al., 2014). Expression and functional characterization of the ARFs are needed if the mechanisms behind how auxin is involved in plant growth and response to environmental stimuli in a spatio-temporal specific manner are to be revealed (Zouine et al., 2014). Auxin has been reported to be an essential regulator of nodulation *via* regulation of mitosis (van Noorden et al., 2007; Plet et al., 2011). Focusing on the main structural and expression features of *ARF* genes in *M. truncatula*, which is a model indeterminate legume, helped us to describe the relationship between ARF-mediated auxin signaling and the symbiotic association with nitrogen-fixing bacteria during the early phase of *S. meliloti* infection.

### Characterization and analysis of the *MtARF* gene family in *M. truncatula*

The reference genome sequence of *M. truncatula*, a forage legume with a small genome size (~500 Mbp) (Young et al., 2011), was used to identify the complete *MtARF* family genes. The number of *M. truncatula ARF* genes was similar to that in *Arabidopsis* (23) (Guilfoyle and Hagen, 2007). Domain analyses are consistent with potential roles as ARFs and provided useful clues for predicting their biological functions. The percentage of CTD-truncated MtARFs (54%) was much higher than the ARF members identified in other plants, such as soybean (15.68%), *Arabidopsis* (17.39%), *B. rapa* (22.58%), rice (24%), and tomato (28.57%) (Shen et al., 2010, 2013; Young et al., 2011; Mun et al., 2012). A typical ARF protein contains a conserved N-terminal DNA-binding domain (DBD), a non-conserved MR and a conserved C-terminal dimerization domain (CTD) (Guilfoyle and Hagen, 2007). ARF protein relies on the DBD to bind specifically to auxin response elements (AuxRE: TGTCTC) in the promoters of auxin responsive genes. The CTD, which is involved in homo- and hetero-interactions among ARFs, resembles the III and IV domain of Aux/IAA proteins (Ulmasov et al., 1997). The presence of a large number of CTD-truncated MtARFs suggested that many auxin-responsive genes in *M. truncatula* can be regulated in an auxin independent manner. ARF proteins function as transcription activators or repressors and are determined by the amino acid composition of MR (Tiwari et al., 2003). The activator/repressor ratio among ARFs in *M. truncatula* was only 0.26, which was similar to tomato (0.27) and nearly half of that in *Arabidopsis* (0.59) or rice (0.56) (Zouine et al., 2014). The data can provide insights into potential functions for *MtARF* genes in plant developmental regulation and responses to environmental stimulus.

### The close relationship between phylogenetic analysis and the expression patterns of *MtARF* genes

Previous studies suggested that phylogenetic analysis provided clues on functional prediction of various genes, including transcription factor encoding genes (Fang et al., 2008; Zhang et al., 2008). The close relationship between sequence conservation and expression patterns helps us to select candidate genes that respond to diverse environmental stimuli, including auxin treatment and *S. meliloti* infection (Wang et al., 2012). Transcript accumulation of most CTD-encoding *MtARF* genes was higher than the CTD lacking *MtARF* genes in the cotyledons, leaves, and stems. It suggested that the CTD protein domain may affect the expression levels of the *MtARF* genes (Figure 3).

In *Arabidopsis*, negative regulation of *AtARF10* by *miR160* participates in seed germination and post-embryonic developmental programs (Liu et al., 2007). As homologous genes of *AtARF10*, the expression of *MtARF1* and *MtARF10* may be also inhibited by *mtr-miR160*. Recently, *miR160* in leguminous plants soybean has been reported by Turner's group (Turner et al., 2013). A set of ARFs repressor could be silenced in plants overexposing miRNA160, suggesting that auxin hypersensitivity was regulated by *miR160* in ARF-dependent manner. Several lines of evidences indicated a role of auxin signaling in nodule development (Bazin et al., 2012). The *miR167* also was found to function in ARF-mediated auxin regulation by reinforcing or maintaining transcriptionally established gene expression patterns (Wu et al., 2006).

*MtARF* gene expression changes respond rapidly to exogenous auxin treatment. A correlation between phylogenetic analysis and auxin-response expression was also revealed by our studies. Most of the *MtARF* genes that belonged to subfamilies I to VII were induced by IAA treatment in the shoots and were down-regulated in the roots (Figure 5). In contrast, most of the *MtARF* genes that belonged to subclass VIII were down-regulated by IAA treatment in the shoots, but induced in the roots. All the results showed that there was a close relationship between sequence conservation and the expression patterns of *MtARF* genes.

### The putative function of MtARFs in nodule formation during the early phase of *S. meliloti* infection

Symbiotic interactions between legumes and rhizobia are required for the formation of nitrogen-fixing nodules, which help soil rhizobia convert atmospheric N_2_ into ammonia for plant absorption (Li et al., 2013). Rhizobial infection and nodule organogenesis are two distinct development processes in nitrogen-fixing nodule formation (Madsen et al., 2010). Many transcriptome and proteome changes occur in both the shoots and roots during the early stage of rhizobial infection, which lead to signal exchanges between the hosts and the bacteria (Godiard et al., 2007; Molesini et al., 2014). A large number of studies have revealed the important roles of auxin in the initiation and development of nodules in different legumes, including white clover, *Lotus japonicas* and *M. truncatula* (Mathesius et al., 1998; Pacios-Bras et al., 2003; van Noorden et al., 2006). In our study, we used *M. truncatula* as a model indeterminate legume to investigate how auxin signaling is involved in nodule formation during the early phase of *S. meliloti* infection.

Being essential components of the auxin signaling pathway, ARFs are likely to bind to the down-stream target genes and function as transcriptional mediators, which trigger appropriate physiological responses in a spatio-temporal specific manner. Most MtARFs have been found to be involved in the responses to the *S. meliloti* infection, which suggests a putative role in the formation of nitrogen-fixing nodules. The expression profile of *MtARF* genes changed significantly during infection and MtARF may activate or suppress the function of many down-stream genes involved in the formation of nodules. Among the 24 *MtARF* genes, only *MtARF1* and 8 were expressed inconsistent with a pattern expected for genes involved in the nodulation process. Interestingly, all the MtARFs that contained a CTD domain belonged to Group 1. It indicated that CTD may be involved in ARF-medicated *S. meliloti* infection responses.

Auxin polar transport inhibitors were used to disturb normal auxin transport and auxin signaling. 1-NOA is an important auxin influx polar transport inhibitor that is used widely in plant physiological studies (Parry et al., 2001). We have test several auxin polar transport inhibitors (including NPA, 1-NOA, and TIBA) in our study (data not shown). The data showed that NPA and TIBA displayed severe inhibition on plant roots growth and elongation and 1-NOA did not affect plant roots elongation too much. Therefore, 1-NOA was used to disturb normal auxin transport and auxin signaling during the early phase of *S. meliloti* infection. The inhibition of auxin signaling by 1-NOA changed the expression profiles of *MtARF* genes during the early phase of *S. meliloti* infection. Interestingly, there was partial similarity between the expression patterns for *MtARF* genes in the mutant *dmi3* and the expression patterns for the wild-type A17 after the 1-NOA treatment. The data indicated that the infection resistant mutant *dmi3* significantly affected the expression of the *MtARF* gene family during the early phase of *S. meliloti* infection. However, the expression patterns of *MtARF* genes under 1-NOA treatment seem to be time (infection)-dependent. In contrast, the expressions of *MtARF* gene attenuations in *dmi3* mutant are less time-dependent, which would be probably due to the insensitivity of the infection. *S. meliloti* infection may first affect the expression levels of *ARF* family genes to help nodule formation and differential expressions of *MtARF* genes may be involved in the resistant phenotype of *dmi3* mutant.

*M. truncatula* is an important model plant for studying symbiosis with nitrogen-fixing bacteria. *MtARF* gene identification, chromosomal location, protein domain analysis, and expression profiling in response to auxin stimulus in different tissues were all investigated in detail in this study. We have provided the expression profiling of *MtARF* genes under *S. meliloti* infection. The involvement of *MtARF* gene expression responses to *S. meliloti* infection was an essential process for auxin signaling functioned in the regulation of nodule formation.

### Conflict of interest statement

The authors declare that the research was conducted in the absence of any commercial or financial relationships that could be construed as a potential conflict of interest.
