# Supplementary material for: Genome-wide identification and expression analysis of auxin response factor gene family in Medicago truncatula
Source: Front Plant Sci. 2015 Feb 24;6:73. doi: 10.3389/fpls.2015.00073 (PMC4338661; doi:10.3389/fpls.2015.00073)
Supplement: Supplementary file 1 [file Table1.DOCX]

| **Table S1 The primer sequences of 24 *MtARF* genes.** | |
| --- | --- |
| ARF1 RTU: | ATTCGTTGGCCTGATTCTCC |
| ARF1 RTL: | CTGGCTCCCTGTATGCTTGA |
| ARF2 RTU: | GCCAACCCAACGAAACAAGA |
| ARF2 RTL: | AATCCCAAAGAGCCTACAGC |
| ARF3 RTU: | CAATCTCAACGACCGCAATG |
| ARF3 RTL: | TCTCCGGCTACGAGTTTCTT |
| ARF4 RTU: | TCAACACCTCCTCGTCACTG |
| ARF4 RTL: | AATGGCTTGTTCTCCTCTGC |
| ARF5 RTU: | GGCATTTGCTCACAACTGGG |
| ARF5 RTL: | AACGCATCCTGAACCTCATT |
| ARF6 RTU: | GCAACCAATAGCCGTTTCAC |
| ARF6 RTL: | TCATCCCAACCAACCTTTAC |
| ARF7 RTU: | CCGATTTCGTTGTGCCTTAC |
| ARF7 RTL: | CCGCAGTTTCTTCAGCCTTC |
| ARF8 RTU: | ATGTGAAGCGAGTAAGTCCA |
| ARF8 RTL: | AGGTGAAGATCCGATAAAGA |
| ARF9 RTU: | CCCTGAAGGAACTGGCAATC |
| ARF9 RTL: | CAGTGTCCGTTGTTGAAGCA |
| ARF10 RTU: | ACGCATCCTCACTCAACCCA |
| ARF10 RTL: | ACCGTCGTCAGCTTCAATCA |
| ARF11 RTU: | TGGCTATGAATGGCGCTTTA |
| ARF11 RTL: | CGCTTACGATGAACTGGCTC |
| ARF12 RTU: | TTGCAGGGTTGTCAATGTCC |
| ARF12 RTL: | CTTCAGCAGCTCTACGAGGC |
| ARF13 RTU: | GTGGCTCTGTTGGAAGGTGT |
| ARF13 RTL: | TGGATGCTTGATTTGGGATT |
| ARF14 RTU: | GGTGCATTCTCAGTGCCTAG |
| ARF14 RTL: | GATCGTTTCGGTTCCAGTTC |
| ARF15 RTU: | ATCTTCCCGCCACTTGACCT |
| ARF15 RTL: | TCCACTGCCTCCGTAAACGA |
| ARF16 RTU: | TGCGAGTGTTGTTGATGAGG |
| ARF16 RTL: | TGGTAATAGAGGCGGTGGAA |
| ARF17 RTU: | CAGCTAGTGGTTGGCGTGAT |
| ARF17 RTL: | AGGAGGAAGTCGAAGGTGCA |
| ARF18 RTU: | TATGGTCCTTCCTTTCCTTG |
| ARF18 RTL: | CAAACTTCCAAACTCGGTCA |
| ARF19 RTU: | CAGCTAGTGGTTGGCGTGAT |
| ARF19 RTL: | TTGAGGAGGAAGTCGAAGGT |
| ARF20 RTU: | TGCGGGTCCCTTAGTTTCTC |
| ARF20 RTL: | ATCACTCGCCGTCAATGTCT |
| ARF21 RTU: | CCGCCACTTGACCTAACATC |
| ARF21 RTL: | ATTCTGCTGCCTCCATAACG |
| ARF22 RTU: | GCCGATGACTGTCCCTAATA |
| ARF22 RTL: | TGTTTCTCCTGCCAATGTTT |
| ARF23 RTU: | CCGTGAAGGCAAACATAACA |
| ARF23 RTL: | AGAAGCGAGAAGCCGAATAG |
| ARF24 RTU: | GACTTGCTTGAATGGTGCTG |
| ARF24 RTL: | TGCCTTTGGTTACCGTAGAA |
| Actin RTU: | CCGTGGAGAAGAGCTACGAG |
| Actin RTL: | TTGGAGCAAGAGCACTGATT |
