# Supplementary material for: Genome-wide identification and expression analysis of auxin response factor gene family in Medicago truncatula
Source: Front Plant Sci. 2015 Feb 24;6:73. doi: 10.3389/fpls.2015.00073 (PMC4338661; doi:10.3389/fpls.2015.00073)
Supplement: Supplementary file 2 [file Table2.DOCX]

**Table S2 *ARF* gene family in *Medicago truncatula***

**Gene Locus ID ORF length No. of Chr Chr Deduced polypeptide**

**(bp) introns no. locations Length (aa) Mol wt (kDa) pI**

***MtARF1* Medtr1g094960 1857 1 1 27028985-27031714 619 68.96 7.52**

***MtARF2* Medtr2g005240 2013 14 2 92538-103811 671 74.98 5.87**

***MtARF3* Medtr2g006270 1368 1 2 510637-513034 456 51.92 8.45**

***MtARF4* Medtr2g006380 969 1 2 557807-558959 323 36.92 6.76**

***MtARF5* Medtr2g014770 2046 10 2 4529157-4533616 682 74.20 6.37**

***MtARF6* Medtr2g018690 2724 13 2 5789327-6794466 908 100.82 6.42**

***MtARF7* Medtr2g093740 2646 11 2 28981107-28985503 882 91.56 6.94**

***MtARF8* Medtr2g094570 2265 5 2 29345021-29348635 755 84.00 8.35**

***MtARF9* Medtr3g064050 2547 13 3 20138634-20145390 849 94.27 6.18**

***MtARF10*  Medtr3g073420 1779 2 3 23919415-23922727 593 64.87 6.41**

***MtARF11* Medtr4g021580 1998 13 4 5935075-5939270 666 47.04 6.39**

***MtARF12* Medtr4g060460 1413 9 4 18652497-18656202 471 53.19 6.82**

***MtARF13* Medtr4g124900 3360 12 4 43700709-43706464 1120 125.42 6.34**

***MtARF14* Medtr5g040740 1230 1 5 17498566-17499946 410 45.10 8.15**

***MtARF15* Medtr5g040880 1572 1 5 17559518-17561643 524 58.20 6.61**

***MtARF16* Medtr5g060630 1569 1 5 24416717-24419362 523 58.09 5.08**

***MtARF17* Medtr5g060780 1131 1 5 24492830-24494398 377 42.09 6.04**

***MtARF18* Medtr5g061220 1563 1 5 24646440-24648245 521 57.87 6.66**

***MtARF19* Medtr5g061890 1131 1 5 24931138-24932785 377 41.92 6.04**

***MtARF20* Medtr5g076270 2523 13 5 31490689-31499972 841 93.28 6.19**

***MtARF21* Medtr5g082140 1380 1 5 34261452-34263293 460 51.65 6.77**

***MtARF22* Medtr7g101280 3756 9 7 32065272-32075513 1252 141.13 8.48**

***MtARF23*  Medtr8g100050 2463 12 8 28958445-28962300 821 91.57 6.43**

***MtARF24* Medtr8g101360 3288 13 8 29291860-29297376 1096 121.07 6.51**
