# Supplementary material for: Genome-wide identification and expression analysis of auxin response factor gene family in Medicago truncatula
Source: Front Plant Sci. 2015 Feb 24;6:73. doi: 10.3389/fpls.2015.00073 (PMC4338661; doi:10.3389/fpls.2015.00073)
Supplement: Supplementary file 3 [file Table3.DOCX]

| **Table S3 Domain positions in 24 MtARF proteins.** | | | | | |
| --- | --- | --- | --- | --- | --- |
|  |  | protein (aa) | DBD (aa) | MR (aa) | CTD (aa) |
| **Medtr1g094960** | MtARF1 | 619 | 0-362 | 363-619 | none |
| **Medtr2g005240** | MtARF2 | 671 | 0-333 | 334-605 | 606-671 |
| **Medtr2g006270** | MtARF3 | 456 | 0-354 | 355-456 | none |
| **Medtr2g006380** | MtARF4 | 323 | 0-323 | none | none |
| **Medtr2g014770** | MtARF5 | 682 | 0-407 | 408-682 | none |
| **Medtr2g018690** | MtARF6 | 908 | 0-376 | 377-767 | 768-908 |
| **Medtr2g093740** | MtARF7 | 882 | 0-428 | 429-668 | 669-882 |
| **Medtr2g094570** | MtARF8 | 755 | 0-410 | 411-706 | 707-755 |
| **Medtr3g064050** | MtARF9 | 849 | 0-388 | 389-727 | 728-849 |
| **Medtr3g073420** | MtARF10 | 593 | 0-359 | 360-593 | none |
| **Medtr4g021580** | MtARF11 | 666 | 0-378 | 379-558 | 559-666 |
| **Medtr4g060460** | MtARF12 | 471 | 0-389 | 390-471 | none |
| **Medtr4g124900** | MtARF13 | 1120 | 0-334 | 335-1060 | 1061-1120 |
| **Medtr5g040740** | MtARF14 | 410 | 0-313 | 314-410 | none |
| **Medtr5g040880** | MtARF15 | 524 | 0-310 | 311-524 | none |
| **Medtr5g060630** | MtARF16 | 523 | 0-349 | 350-523 | none |
| **Medtr5g060780** | MtARF17 | 377 | 0-314 | 315-377 | none |
| **Medtr5g061220** | MtARF18 | 521 | 0-339 | 340-521 | none |
| **Medtr5g061890** | MtARF19 | 377 | 0-314 | 315-377 | none |
| **Medtr5g076270** | MtARF20 | 841 | 0-386 | 387-719 | 720-841 |
| **Medtr5g082140** | MtARF21 | 460 | 0-315 | 316-460 | none |
| **Medtr7g101280** | MtARF22 | 1252 | 0-385 | 386-659 | 660-1252 |
| **Medtr8g100050** | MtARF23 | 821 | 0-702 | 703-765 | 766-821 |
| **Medtr8g101360** | MtARF24 | 1096 | 0-390 | 391-988 | 989-1096 |
