# Supplementary material for: Genome-wide identification and expression analysis of auxin response factor gene family in Medicago truncatula
Source: Front Plant Sci. 2015 Feb 24;6:73. doi: 10.3389/fpls.2015.00073 (PMC4338661; doi:10.3389/fpls.2015.00073)
Supplement: Supplementary file 5 [file Image1.PDF]

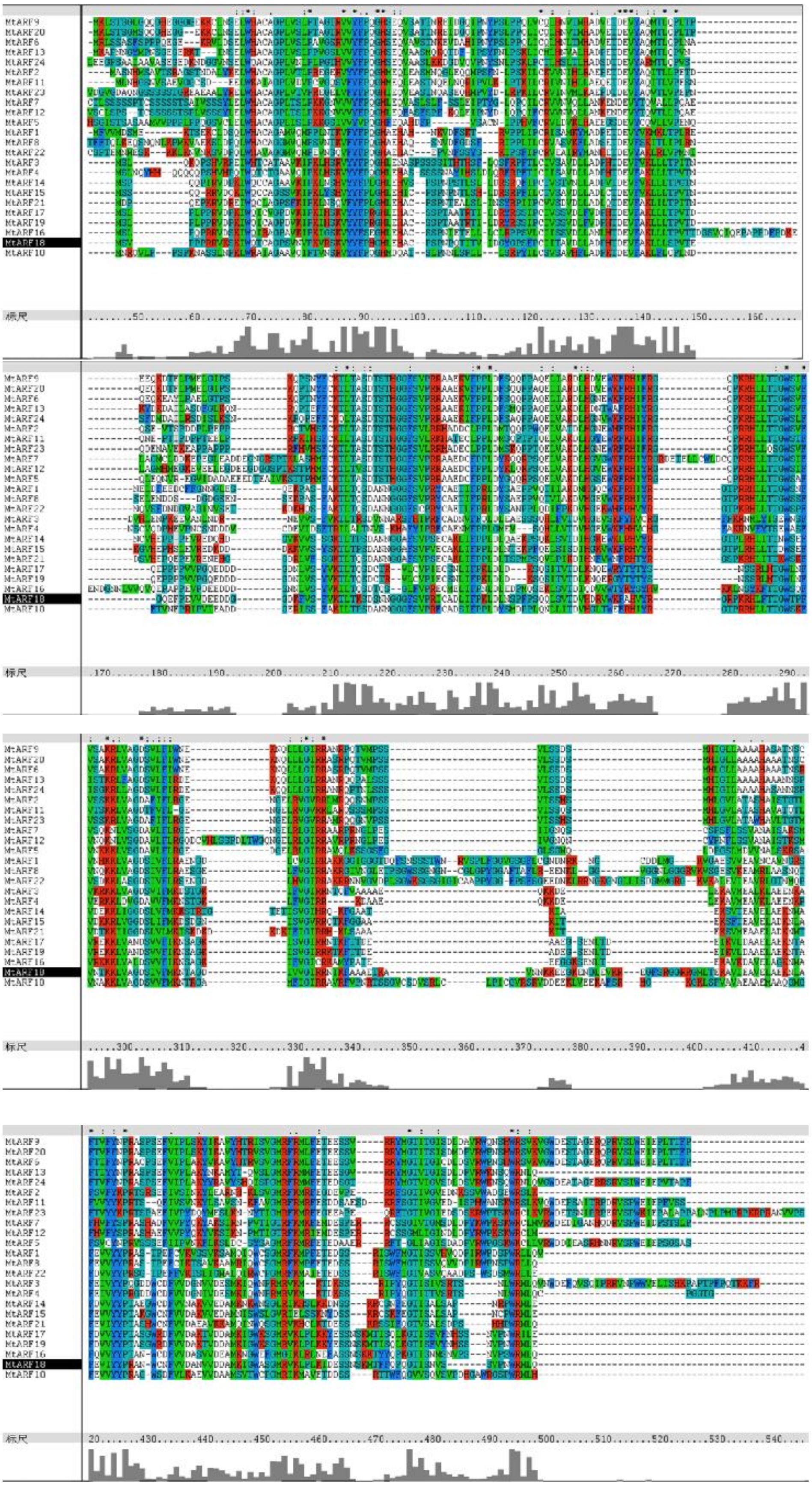

Figure S1. Multiple alignment profile of DBD domain of MtARF proteins obtained with ClustalW program.
