# Supplementary material for: Genome-wide identification and expression analysis of auxin response factor gene family in Medicago truncatula
Source: Front Plant Sci. 2015 Feb 24;6:73. doi: 10.3389/fpls.2015.00073 (PMC4338661; doi:10.3389/fpls.2015.00073)
Supplement: Supplementary file 6 [file Image2.PDF]

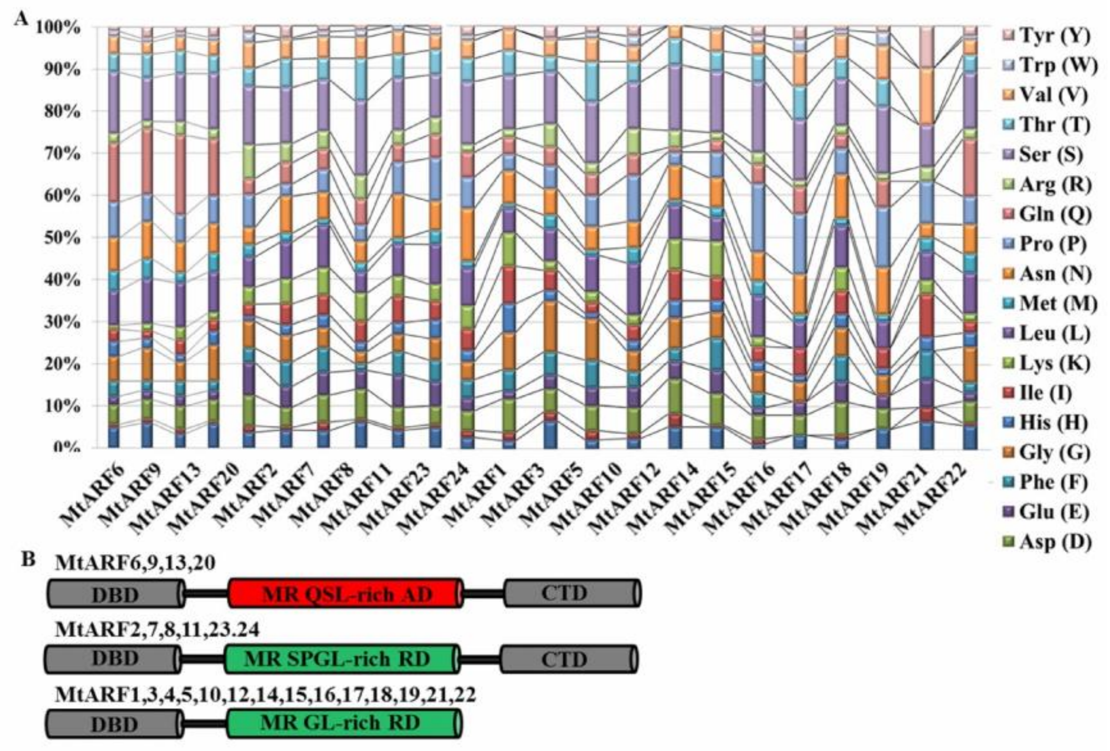

Figure S2 Analysis of amino acid contents and classification of MtARF proteins. (A) Amino acid contents of MR domains in putative MtARF proteins. MtARFs are indicated by the horizontal axis, and the corresponding amino acid contents are indicated by the vertical axis. Colour bars represent the different amino acids. The positions of different domains in MtARF proteins are listed in Table S1. **(B)** The protein structure of MtARF family. DBD, DNA-binding domain; CTD, C-terminal dimerization domain; MR, middle region; RD, repression domain; AD, activation domain; Q, glutamine; S, serine; L, leucine; P, proline; G, glycine.
